# Collectanea Medica

**Published:** 1814-09

**Authors:** 


### COLLECTANEA MEDICA,

CONSISTING OF

ANECDOTES, FACTS, EXTRACTS, ILLUSTRATIONS, QUERIES, SUGGESTIONS, &c.

RELATING TO THE

*History or the Art of Medicine, and the Auxiliary Sciences.*

---

### *Case of Hydrophobia which terminated fatally.* By Mr. H. Marshall, Surgeon, Colombo, Ceylon.

MONDAY, the 27th April, a favourite little spaniel dog, belonging to Mr. D. was bitten by a dog running along the street where he resided. On the 2d May, about 9 A. M. Miss D., a young lady about fifteen years of age, was, without giving any provocation, bitten by her father's spaniel. The teeth of the animal penetrated the skin of the palm of the right hand, and lacerated the flexor muscles of the thumb. During the course of the same day, this dog bit also three of Mr. D's servants. The dog was observed to eat and drink as usual, until the evening of the 3d, when he was confined, although nothing alarming had then taken place in his appearance, nor was his manner observed to be materially changed. On the morning of the 4th, he very evidently evinced symptoms of uneasiness, which the spectators, with too much reason, suspected were indications of madness: meat and drink were now refused. He died on the same day about one o'clock.

The lacerations in the hand of Miss D. were dressed with blistering plaster, and occasionally a few drops of eau de luce were exhibited. On the 4th May, about noon, I was requested to visit her. At this time, I removed the lacerated skin and muscles; her hand was immersed in warm water for nearly an hour, and then lunar caustic was liberally applied to the whole extent of the wound. Two of the servants who were bitten were treated in the same manner; the third had caustic only applied to the wounds; as he had received the injury in the groin, too near the large blood-vessels to render an attempt at complete excision adviseable. The following day I visited the family of Mr. D. when my attendance for that time ceased. Miss D. was daily visited by a native practitioner, and, from his narrative, together with her father's information, I have collected the following particulars.

Caustic was occasionally applied to the wounds, until the 15th May. She commenced to take calomel on the 20th, and continued that medicine until the 3d June. Salivation was quickly produced, and continued for a considerable time after she ceased to take calomel.

On the 9th May, the catamenia appeared, and they recurred on the 14th June: this day she was very unwell, and complained of great languor—in the evening she took some chamomile tea, which operated powerfully as an emetic. She perspired profusely during the greater part of the night. On the 15th, she came to the breakfast-table, but had no appetite, and ate nothing. When water was brought to her to wash her mouth, she was observed to tremble. About noon, some arrow-root was dressed, which she requested should be given to her in a coffee-cup, and not in a glass, as her friends intended; she shivered strongly when she saw the arrow-root. She succeeded, however, in swallowing a little of it, although with infinite difficulty. At dinner she ate a little, but now very evidently shuddered when water was brought to her view. She swallowed a little tea, and ate a small portion of bread, about five P.M. At seven she swallowed a little coffee; in an hour after, she perspired very profusely, and complained of great giddiness. Between nine and ten o'clock, the doors of her room were opened to admit more air; she instantly complained of the current of air giving her great uneasiness, and begged to be removed from it. She often attempted to sleep during night, but was almost instantly awakened by frightful dreams, hastily starting up, trembling, and shivering. About three A.M. she complained of a sensation of extreme heat in her throat and belly; the giddiness at the same time continued unabated. At her own desire, water was brought to her bed-side to bathe her feet; on her perceiving it, she shuddered strongly, and turned from it as if terrified. In a short time, she so far conquered her aversion to water, as to place her feet in the tub for a few minutes; for a little time she thought the giddiness relieved. I was called to visit her on the morning of the 16th, and reached her house about half past seven o'clock, where I was soon joined by Drs. Anderson and High. We found her sitting on a bed in a reclining position, supported by pillows, and her arms placed across her body. The muscles of the face were frequently convulsed, and the angles of the mouth occasionally drawn back. Her eyes were glassy, and at intervals fixed with a wild unmeaning stare. The collection of saliva in the mouth gave great uneasiness, although it was partially expelled by expiration in a state of foam. Every three or four minutes the viscid phlegm seemed to irritate and throw into convulsions the muscles of the throat, when she exerted her whole force to expel the offending fluid.

The skin was covered with perspiration; it ran over the face in drops. Her pulse was about 100, and sharp. Her respiration was hurried and unequal. The countenance expressed great anxiety; occasionally it indicated extreme agony. She sometimes glanced her eyes through the room; while her countenance expressed a suspicious watchful dread of some impending evil. Except when the irritation and spasms of the muscles of deglutition occurred, the chief uneasiness she complained of was a painful sense of heat in the throat. The giddiness of the head also continued to trouble her. In general she seemed unwilling to speak, and when she attempted it, the tongue faltered, the lips trembled, and her lower jaw did not readily obey the will. Her speech was consequently hurried, and not always very intelligible. She seemed sensible and perfectly conscious of what was passing around her; she expressed an earnest desire that her father and sister should remain constantly by her. At her own request, a little little rice was brought, of which she swallowed a small portion, but after an extremely painful exertion of the muscles of the throat. On hearing one of us express a wish to see water brought into her view, she readily complied, and showed great willingness to gratify us, although she was evidently afraid to make the trial. She even put a cup which contained some water to her lips, and attempted to swallow a little, which was instantly rejected with great force by the spasmodic constriction of the muscles of deglutition; this attempt was also accompanied with general convulsions. When water was brought in her view a short time after, she turned from it, with a countenance expressive of horror.

When the hand was examined, it was found that the wound was perfectly healed, leaving a slight eschar in the skin, but without induration or tumefaction of the parts under it.

She was bled in the right arm by a large orifice, and the blood permitted to flow until faintness was produced; the quantity of blood taken away was about twenty-four ounces. Soon after the vein was opened, she very intelligibly expressed herself generally relieved; particularly of a heaviness and giddiness of the head. The spasms of the muscles of the throat were evidently less violent, and also much less frequent; her countenance became comparatively placid and natural. Before the vein was closed the pulse was so weak as scarcely to be felt at the wrist. During her state of faintness, a vial containing eau du luce was applied to her nose, which instantly produced strong involuntary actions of the muscles of the throat and face.

In about ten or fifteen minutes after the arm was tied up, the morbid symptoms returned, with increased violence; debility supervened rapidly. An enema, with four drachms of the tinct. of opium, was now exhibited. Shortly after, she was suddenly seized with a severe pain and sensation of burning in the regions of the epigastrium and umbilicus, in consequence of which she shrieked out in great agony. She continued to scream for about twenty minutes, and then became suddenly quiet. A warm bath having been previously prepared, she was now put into it. She expressed no horror either at the sight of the water or on immersion. After being removed from the bath, she was put to bed. The system now seemed extremely relaxed and debilitated; the pulse could not be felt at the wrist; the pupils of the eyes were dilated, the countenance was sunk, and the skin covered with a cold perspiration; foam was no longer formed at the mouth, —probably the power of swallowing returned. Her breathing was hurried, and accompanied with a convulsive sob. At a quarter past nine A. M. she expired.—*Edin. Med. and Surg. Journal.*

---

### *Case of Tic Douloureux, cured by the external use of Tar.* By Mr. E. Colville, Surgeon, Ayton.

Mr. J. L. consulted me on the 8th of September last, on account of a severe pain in the left cheek and temple. It began about four years before that, without any apparent cause, and continued, with very little intermission. It seized him in paroxysms, which at last came on every three minutes. Whenever he attempted to eat or drink he was seized with dreadful pain; the tears gushed from his eyes, and he involuntarily pressed the part with both hands, with as great force as he could. He was deprived of his rest during the night; and in consequence he felt himself very feeble and frequently sick. His belly has been pretty regular, but when I saw him he was rather costive. He had been of very regular habits all his lifetime; his age about 72 years.

As his pulse was rather quick, I took eight ounces of blood from the left temporal artery, gave him some doses of sulphate of soda, to take one when necessary, and pills, each containing one-fourth of a grain of white oxide of arsenic and half a grain of extract of hyoscyamus, one of which he took evening and morning, and an embrocation of tinct. sapon. c. opio, to rub on the pained parts, and drop into the ear. I saw him again three weeks after. The pain was not in the least degree relieved, except for a few minutes after he dropped the tincture into the ear. The sickness had also been in a great measure relieved, which he attributed to having taken the salts regularly. By his own desire I extracted three stumps from the lower jaw; and, as the pills seemed to have had no effect, I ordered them to be discontinued. I ordered him to take a dose of salts every third day, and did not see him again until the end of October. His health was then pretty good, but the pain not in the least abated. I proposed the operation of dividing the nerve, which he would not agree to on account of his age.

I saw him again on the 9th of February this year, and was surprised to find him quite well. He told me that, in consequence of his daughter having found great relief from tar as an external application to rheumatic pains in several parts of the body, by my recommendation he was advised to try it for his disease, which he did about the beginning of January. He rubbed a little upon the cheek and temple for three nights successively. He felt no relief from it the first night; on the second the pain was rather easier; and, on the morning after the third rubbing, the pain was quite gone, and he has never had the slightest paroxysm since, and is as well as he has ever been in all the course of his life. I think there can be no doubt that this was a case of Tic Douloureux. The pain indeed extended farther upon the temple than it generally does; but all the symptoms taken together make the case quite plain. It attacked in paroxysms, which were always severest when he attempted to eat; great nervous irritation; pulse a little quickened; face frequently flushed during the paroxysm; the muscles of the mouth frequently contracted, causing considerable distortion of the face on the left side, &c. I should be happy to hear if any gentleman meets such a favourable result from a trial of the remedy.—*Edin. Med. and Surg. Journal.*

---

*Case of Rheumatism of the Heart.* By J. PENKIVIL, Esq. Surgeon, Plymouth Dock.

Mrs. W. a thin delicate woman, about 50 years of age, was seized with general symptoms of pyrexia, as I learned from her friends friends, a few days before I was sent for. On my first visit, I found her much prostrated in strength, her skin having febrile heat, the pulse rather below the standard in point of number, yet remarkably full, with a vibrating wirey throb, conveying the idea of inflammatory action. The tongue was white and coated; the bowels rather costive; the ankles were swelled and painful, though not red; the knees were much more swelled, and so painful, that she could not bear the slightest touch without crying out; but the most distressing symptom was an excruciating pain over the left temple, with violent throbbing and a hissing noise, which rendered her deaf on that side. The remarkable prostration of strength, in a frame otherwise very delicate, deterred me from having recourse to general bleeding. I therefore preferred local bleeding, by leeches applied to the painful temple, and a blister applied to the back of the neck. As it was then night, I ordered a sudorific draught to be taken immediately, and a dose of purgative pills to be taken early the next morning; sinapisms were also ordered to the painful joints. The next day she experienced some relief from the remedies applied to the head and temples; but the joints of the inferior extremities continued equally painful, and those of the superior began to take on a similar action, particularly the hands. The opening pills, which had produced their usual effect, were repeated; powders, with potass. nitrat. were ordered three or four times a day, together with antimonials, and the joints affected rubbed with ol. camph. This plan was followed, in a few days, with an alleviation of some of the symptoms, but an aggravation of others; the joints of the inferior extremities grew better, whilst those of the superior became worse. The violence of the pain of the temple, it is true, was somewhat abated; but the hissing noise remained, though I think she was not quite so deaf. Great watchfulness now supervened, with remarkable anxiety at the præcordia, so that I found it necessary to exhibit an opiate combined with an antimonial at night; the powders were changed for a camphorated mixture, to which was added some liq. ammon. acet. given every fourth hour. A little more rest and ease was procured in consequence of this alteration; but now the complaint, which so far may be considered to have been purely rheumatismus acutus, with the addition of the peculiar affection of the left temple, assumed a change of symptoms. The inflammatory action seemed to fly, as it were, from the circumference to the centre. The heart and arterial system became peculiarly affected; their pulsations were slower than natural, but remarkably full and violent, so that the action of the carotids could be seen almost at any distance of the room, and when the hand was applied to the region of the heart, it seemed by its force to be lifted from the part. This violent action was now and then followed by a sudden collapse, consequent faintings, and slight twitchings. It will be expected now that the pain of the temple, and the hissing noise from the force of the carotids and temporal arteries must have been increased. The case assumed a serious aspect. I therefore drew about ℥xvi. of blood from the arm, and the bowels being previously well purged, ordered an anodyne sudorific draught. The next morning the assistance of Dr. Woollcombe was requested. The blood appeared somewhat, not particularly, sizy, but certainly the patient was relieved by its extraction. The doctor's attention was particularly struck by the violent arterial action, and, on turning down the bed-clothes, our patient being very thin, the systole and diastole of the abdominal aorta were evident to the eye. He was equally surprised at the slowness of the pulsation, which at that time was not much above 40 in a minute. Having recommended the left side of the head to be previously shaved above the painful temple, he ordered a blister to be applied there, and prescribed an eight-ounce mixture, similar to that I had previously sent, but adding to it ℥i. vin. antim. tart. of which a fourth part was to be taken every sixth hour, with a powder containing xv. gr. jalap, until the bowels were freely opened. A draught, with æther t. lav. c. and t. opii was to be given at night after the operation of the powders, especially should the watchfulness continue. The next morning, the pain of the temple was much relieved by the application of the blister, and the general arterial action was moderated, perhaps by the blood previously drawn, but also, I think, by the purgatives and vin. antim. tart. now prescribed. As the action of the heart and arteries had a little subsided, so their pulsation had risen a little nearer their standard. Indeed it is observed, that the pulse gained in velocity what it abated in force, gradually, as the patient recovered, approaching the natural standard. Following this plan of treatment, Mrs. W. in the course of a week, was convalescent, every symptom vanishing, together with the deafness, except slight stiffness and occasional shootings in her joints, which in another week or two left her; and I have lately had the pleasure of seeing her in perfect health.—*Edin. Med. and Surg. Journal.*

---

*Report of the Progress of the Sciences in France in* 1813; by J. C. Delametherie.

*Chemistry.*—In the chemical analysis of animal substances Berzelius has made great improvements. He has submitted the animal fluids, and particularly the blood, to new analyses. The blood, he says, is composed of two parts; a liquid, the *serum*, and one which is suspended, the *coagulum*. In the animal œconomy we ought to distinguish three principal substances: *a*, fibrine; *b*, albumen; *c*, gelatine.

The serum according to Berzelius, is a solution of a great quantity of albumen with a little *fibrine.* Both are combined with soda. It also contains some other saline substances.

The coagulum is the colouring matter. It differs from albumen in its insolubility in serum, and by its colour. The colouring matter amounts to about one-third of the mass: the colour seems to be owing to iron, of which it contains about one-third of its weight; but this iron can be separated by combustion only.

This colour cannot be produced artificially by uniting albumen

 with with subphosphate of iron, as Fourcroy and Vauquelin have asserted. Nor is it possible to produce it by uniting iron with soda, as Parmentier and Deyeux have supposed.

We may compare the colour of the blood to the other red colouring principles formed by animals, cochineal, kermes, the purple of the murex, &c.

Four hundred grains of colouring matter, when incinerated, yielded:

| | Grains. |
|---|---|
| Oxide of iron | 30· |
| Subphosphate of iron | 7·5 |
| Phosphate of lime and a little magnesia | 6· |
| Pure lime | 20· |
| Carbonic acid and loss | 16·5 |

The serum of the blood gave upon analysis:

| | |
|---|---|
| Water | 90·5 |
| Albumen | 80· |
| Muriate of potash, soda, and lactate of soda | 4· |
| Soda, phosphate of soda, and a little animal matter | 4·1 |

In speaking of the lactate of soda, Berzelius observes, that the existence of the latic acid discovered by Scheele had been erroneously doubted by Fourcroy and Vauquelin. The blood containing no gelatine nor earthy phosphate.

The fibrine, albumen, and colouring matter resemble each other so closely, that they may be considered as modifications of one and the same substance: they give earthy phosphates and carbonates of lime solely when they are decomposed.

The author thinks that the phosphate of iron does not exist in the non-decomposed colouring matter, and that which we obtain by incinerating it is a product of combustion.

The colouring matter dried and exposed to the fire in a red-hot crucible melts, swells, and burns with a clear flame; it leaves a pory charcoal, which burns with difficulty. In burning, a smell of ammonia is constantly exhaled, although it had been exposed several times to a strong fire.

This extrication of ammonia from a burning charcoal, and which has been long exposed to the fire, is according to him a remarkable phænomenon: hence he concludes that this ammonia is a new product.

---

*On the Extrication of Caloric during the Coagulation of the Blood*; by JOHN GORDEN, M. D. F. R. S. E. and Lecturer on Anatomy and Physiology, Edinburgh.

*Edinburgh, July* 1, 1814.

MY friend Dr. John Davy, in his interesting thesis (Quædam de Sanguine Complectens) printed here last month, has alluded to an experiment of mine, relating to the extrication of caloric during the coagulation of the blood, which I have been accustomed to mention in my anatomical and physiological lectures for three years back.

back. In the lectures on physiology which I delivered during the three summer months of last year, the limited period of the course did not permit me to dwell so long on that, nor on many other subjects, as I could have wished. To this, I doubt not, it is to be attributed, that the experiment referred to has been in some degree misapprehended by Dr. Davy; and, if by him, who honoured me with the most flattering attention during the whole of that course, I fear also by many other of my pupils.

The following, however, are the notes relating to this subject, from which I then read:—

"As it is a fact established in chemistry that the conversion of a fluid into a solid is always accompanied with an extrication of caloric, one could have little doubt, even if the fact were not made evident by experiment, that the caloric is extricated during the coagulation of the blood.

"Fourcroy had stated it, as the result of experiments made by him at the Paris Lyceum, in 1790, that during the coagulation of bullock's blood as much caloric was given out as raised the thermometer (Reaumur's I presume,) five degrees. (Ann. de Chimie, t. vii. p. 147.)

"But more authority seems to have been attached by physiologists to the following experiment of Mr. John Hunter. Mr. Hunter having suspended a healthy turtle by the hind legs, cut off its head, and caught the blood in a bason. The blood while flowing was 65°, and when collected was 66°, but fell to 65° while coagulating, which it did very slowly. It remained at 65°, and when coagulated was still 65°. From this, and similar experiments, Mr. Hunter concluded that in the coagulation of blood no heat was given out. (Treatise on the Blood, &c. 4to. p. 27.)

"A very different result, however, has since been obtained by the author of a short article on the blood in Rees's Cyclopædia. Ten ounces of blood was drawn into a wooden bowl, in which a thermometer was held. The temperature of the blood while flowing from the vein was 93°. In six minutes the thermometer had sunk to 89°, and coagulation commenced on the surface. On elevating the bulb of the thermometer to the coagulum on the surface, the mercury rose to 90½°, and on again depressing it to the bottom of the bowl it sunk to 89°. This was repeated twice, with nearly the same result; and on the third trial the quicksilver rose to 91°. The blood was now coagulated throughout; and, after this, the mercury continued to descend regularly, and was no longer influenced by changing the situation of the bulb of the thermometer. In this experiment it clearly appeared that during the coagulation of the blood caloric was extricated; and in sufficient quantity, at one time, to raise the thermometer 2°.

"As it was desirable to confirm this result, and, of course, that before obtained by Fourcroy, both so comformable to analogy, my friend Mr. Ellis and myself, in presence of Professor Thomson, performed the following experiment, in the month of April, 1810:

"Blood was received from the femoral artery of a dog, into a small small glass jar. The temperature of the blood flowing from the artery was 99° Fahr.; and that of the apartment, during the experiment, 46° Fahr.

"One minute after the blood had been received into a vessel, it began to coagulate, by a film on the surface. The bulb of a very delicate centigrade thermometer was now placed into the blood at the upper part of the vessel, and held there during a minute, without touching the sides of the glass. It was then depressed to the lower part of the vessel, where coagulation had not begun, and held there in the same manner during the next minute. During the next it was held at the top, and during the next at the bottom; and so on, it was alternately elevated and depressed for 20 successive minutes after coagulation had begun on the surface.

"When the bulb was first placed in the blood at the top, the mercury gradually rose to 34°; but, when it was depressed towards the bottom, it instantly fell to $30\frac{1}{2}$°. When again elevated, it rose to $33\frac{1}{2}$°; and, when again depressed, sunk to 30°. A third time brought to the surface, the mercury rose to 32°; and a third time depressed, it fell, in half a minute, to $28\frac{8}{10}$°. At the next elevation the mercury rose to 31°; and at the succeeding depression, fell to $28\frac{1}{2}$°. At $18\frac{1}{2}$ minutes after the blood had been drawn, when the bulb of the thermometer was brought from the bottom towards the top, the mercury rose from 24° to $25\frac{1}{2}$°. It was now held at the top for two minutes, and the mercury gradually fell to 24°. The blood seemed now completely coagulated, and the experiment was discontinued.

"In this experiment, therefore, the extrication of caloric during the coagulation of the blood was rendered sensible by the thermometer for 20 minutes after the process had commenced; and was at one period so great as to raise the thermometer, in this cool apartment, $3\frac{1}{2}$° of the centigrade scale, which is equal to 6·3° Fahr.

"That a similar extrication of heat was not apparent in Mr. Hunter's experiment on the blood of the turtle, may have been owing to his not having placed the bulb of the thermometer alternately in the coagulating and yet uncoagulated part; and partly, perhaps, to the slowness with which he informs us the process went on.

"We may regard it, therefore, as established, that, when part of the blood thus spontaneously passes from a fluid to a solid state, caloric is extricated, in the same manner as when other fluids undergo a similar change."

Farther than this, my notes did not then extend.

But during last winter I had an opportunity of trying this experiment several times, on venous blood drawn from persons labouring under inflammatory complaints, and the result was always precisely similar. The following is a note of one of these experiments:—

Jan. 22, 1814. I received three ounces of blood from the median vein of a man, aged forty, labouring under pneumonia, into a tall glass vessel, and immediately introduced into it a delicate Fahrenheit thermometer, the bulb touching the bottom. The temperature of the blood in this situation was 76°. In two minutes fluid *size* collected at top; and in two more a very thin film appeared on the surface of this size, and the thermometer was then exactly 74°. In four minutes more, that is, eight minutes after the blood had been drawn, a soft coagulum was formed to the depth of an inch from the top, and the thermometer (the bulb being still at the bottom) was 73°. I now raised the bulb smartly into the middle of this coagulum, and instantly the mercury rose to 85° (that is, 12°); and when I depressed it again to the bottom, where the blood was still fluid, the mercury immediately sunk to 73°. I repeated this several times with similar success. The temperature of the apartment during the experiment was 55°.

I have tried this experiment again, within these few days, on blood drawn from the arm of a middle aged man, labouring under an affection of the heart, and the result was similar.

On carefully reviewing all these experiments, I cannot discover any source of fallacy connected with them, which should lead me to hesitate in deducing from them the same conclusion as formerly, viz. that caloric is extricated during the coagulation of blood, and therefore that the blood is no exception to the general law applicable to all other fluids in this respect.

That my friend Dr. Davy has been led to adopt an opposite opinion in consequence of his experiments on lamb's blood, has obviously arisen from his not having been aware of the importance of moving the thermometers in his experiments;—a circumstance for a knowledge of which I am myself entirely indebted to the anonymous author already referred to in Rees's Cyclopædia; and a circumstance, I may add, which, unless it be scrupulously attended to, is calculated to render all experiments made with a view to ascertain the temperature of blood after it has been drawn from the vessels, altogether inconclusive. At the same time, I cannot help observing, though with the utmost deference to one so much more familiar with chemical details than I am, that, taking Dr. Davy's experiments as they are, they rather seem to me to warrant an opposite conclusion to that which he has drawn from them; and that, upon the whole, they rather tend to confirm than to disprove the extrication of caloric during the coagulation of the blood.—*Annals of Philosophy.*

CRITICAL

